# Investigating the Mechanical Properties and Temperature Compensation of a Spot-Welded Strain Sensor within an Intelligent Steel Strand Cable

**DOI:** 10.3390/s24030745

**Published:** 2024-01-24

**Authors:** Nianchun Deng, Lehai Hu, Xin Liu, Zhiyu Tang

**Affiliations:** 1 College of Civil Engineering and Architecture, Guangxi University, Nanning 530004, China; dengnch@gxu.edu.cn (N.D.); hulh1009@163.com (L.H.); 2310391069@st.gxu.edu.cn (X.L.); 2 Guangxi Key Laboratory of Disaster Prevention and Engineering Safety, Nanning 530004, China

**Keywords:** intelligent steel strand cable, spot-welding strain sensor, cable force, strain transfer, temperature-sensitive characteristics

## Abstract

According to current regulations, welding is strictly prohibited for prestressed and tension cables. In response, this article proposes the use of a portable spot-welding machine to spot weld steel strands. This method generates a small current during spot welding, with a voltage of only 3 V to 5 V, and does not damage the internal structure of the steel strand. To effectively monitor cable tension in cable-supported structures, a novel approach utilizing a chip-based, encapsulated spot-welded strain sensor was investigated. The strain sensing capability, temperature sensitivity, stress relaxation, and static load responses were investigated on the proposed smart steel strand cables with spot-welded strain sensors. The theoretical analyses and finite element simulations revealed that the strain transfer efficiency of the spot-welded strain sensor exceeded 96%. The experimental results demonstrated that the load-strain relationship of the smart steel strand cable had a fitting degree greater than 0.999, and the tension errors obtained under different loads were within 1.26%. The tension full capacity errors measured at different temperatures were generally within 1.0%. The relaxation rate of the smart steel strand cable after 120 h was 3.78% and reduced the sensor accuracy error by 3.97%. Thus, the proposed strain sensor equipped with a smart steel strand cable is suitable for use in long-term tension monitoring.

## 1. Introduction

A bridge cable is an important load-bearing component of cable support bridges (including sling arch bridges, cable-stayed bridges, and suspension bridges). The durability and reliability of the entire bridge system are directly related to the operational safety during the service lifetime of the bridge. However, most bridge cable conditions are poor; almost all in-service bridge cables exhibit different degrees of disrepair and deterioration, and bridge collapse accidents caused by cable fractures occur occasionally [1,2] The continuous monitoring of the force conditions of cable systems is therefore necessary to minimize accidents [3,4]. Achieving this task requires the consideration of the installation survivability, stability, and durability of the monitoring devices. However, contemporary sensor-based monitoring devices often exhibit a poor installation stability and fail to satisfy long-term monitoring requirements. Given their unstable post-installation status, frequent maintenance is required to ensure stability. This process involves significant human resources. Therefore, adopting a reliable and effective test method to realize cable force monitoring of cable-supported bridges and to monitor the health and operating status of bridge cables is highly relevant [5,6].

Currently, the cable force measurement methods mainly include the vibration frequency method, resistance strain gauge method, vibrating wire pressure sensor method, resistance strain pressure sensor method, hydraulic pressure sensor method, magnetic flux detection method, grating fiber reinforced polymer (FRP) smart tendon method, and grating implantation grooved steel wire method [7,8,9]. The vibration–frequency method determines the cable force by considering the frequency and various cable parameters in a cable force equation. In its theoretical derivation process, the boundary conditions of the cables are simplified; for example, cable vibrations are considered without damping. However, it is difficult to satisfy all these assumptions in practical scenarios, where parameters such as the bending stiffness of the cable have a significant influence on the resulting cable force [10,11,12]. In [13] a vibrating wire pressure ring, strain pressure ring, and hydraulic pressure sensor were installed at the end of a cable. However, considering the critical influence of friction between the cable and the embedded pipe on the measured value, such methods are rarely used in practical scenarios. A magnetic flux sensor has favorable application prospects and is suitable for measuring a static cable force with a measurement accuracy of 3–8% [14]. Fiber Bragg grating intelligent strand cables and strain gauge-equipped intelligent steel strand cables [15,16,17] have not been promoted on a large scale, because it is difficult to paste fiber gratings and strain gauges on steel strands; moreover, the problem of detachment due to glue aging [18] also persists. The resistance strain gauge method uses foil-type strain gauges pasted on the steel wire in the cable body to measure the external force of the cable; the sensitive grid wire is deformed to change its resistance to output an electrical signal, and the cable force is obtained via data analysis and processing of the information recorded by the strain collector.

Compared with other measurement methods, the resistance strain gauge method was developed first and remains widely used. This strain gauge method offers the advantages of a small size, low cost, relatively simple installation process, and high measurement sensitivity. However, the resistance strain gauge method is limited by challenges in terms of the adhesive curing temperature range and prolonged curing time. Field experiments have shown that more than 24 h are often required for an adhesive to be fully cured, rendering real-time monitoring impractical. Furthermore, the long-term application of resistance strain gauges on steel wires is challenging because the adhesive may deteriorate over time, leading to detachment. Therefore, when using resistance strain gauges to measure the cable tension in steel strand cables, it is essential to consider the effective integration of the gauges within the cables to ensure long-term stable monitoring. To develop a reliable and practical testing approach for cable-supported bridge tension monitoring during construction and the subsequent monitoring of bridge operation, a self-perceptive monitoring method involving spot-welding strain sensors onto steel strands or wires is proposed for the formation of smart cable structures. This approach facilitates an immediate measurement after welding, resulting in a simple and efficient with strong connection performance at the welding points [19]. By employing spot welding for sensor installation, a high survival rate of strain sensors in smart cables can be achieved, thereby enhancing the accuracy and precision of tension measurements. This methodology has significant value for engineering applications. The corresponding structure has a great influence on the temperature of the strain test, making it problematic to directly compensate for the temperature strain of the measuring point with the temperature strain of the compensating block. Due to the effects of other factors, the temperature strain at the measuring point is not equivalent to that at the compensating block [20]. This article first introduces the principle and fabrication of spot-welded strain sensors. Then, tests are described considering the welding strength and strain transfer of spot-welded strain sensors, and experimental scenarios are simulated using finite element modeling. Finally, the spot-welding strain sensor is implanted into the cable, the sensing performance and temperature sensitivity of the smart cable are tested, and a corresponding temperature compensation method is developed.

## 2. Principle and Package of the Spot-Welding Strain Sensor

### 2.1. Basic Principle of the Spot-Welding Strain Sensor

Strain electrical measurements include the technique of applying electrical methods to measure strain. These approaches offer high measurement accuracy and convenient application while facilitating the performance of strain measurements on large-scale engineering structures in various and complex working conditions. When measuring the strain of the test piece, the strain gauge needs to be pasted on the test section according to the measuring point diagram. If the section deforms with the action of load F, then this value is related to the sensitivity grid of the strain gauge through the basement of the strain gauge and its adhesive, and the sensitivity grid changes the resistance, thereby allowing the conversion of nonelectric strain into electric resistance. Suppose that the resistance of the strain gauge is *R*_0_, the sensitivity coefficient is *k*, and the resistance change caused by the deformation of the test piece is Δ*R*. The relationship between the resistance change rate of the strain gauge and the strain *ε* can be expressed as follows:

(1) $$∆R/R_{0}=k\varepsilon$$

**Figure 1:**
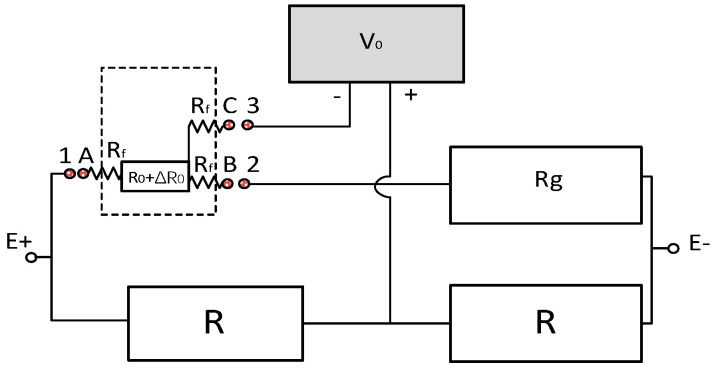
Three-wire connection diagram.

Currently, the most widely used strain electrical measurement method in engineering is based on the Wheatstone 1/4 bridge circuit. According to this method of connecting the bridge circuit to the different measuring equipment, the strain measurements are divided into two-wire and three-wire systems. However, electromagnetic interference or temperature differences greatly affect two-wire systems. Reference [21] proposed a three-wire bridge circuit, as shown in Figure 1, where E is the excitation voltage, R is the voltage divider resistance, *R*_0_ is the strain gauge, and *R_g_* is the matching resistance (*R*_0_ ≈ *R_g_*). Then, the strain gauge used three lead *R_f_* values of A, B, and C to connect the ports 1, 2, and 3 of the device. If the test section is deformed, then the resistance value of the strain gauge *R*_0_ will change accordingly with respect to Δ*R*. The bridge circuit voltage measured by the device is *V_t_* at this moment, which is given as:

(2) $$V_{i}=(\frac{R_{0}+∆R+R_{f}}{R_{0}+∆R+R_{g}+2R_{f}}-\frac{R}{R+R})$$

If the test piece is not deformed, when ∆*R* = 0, *R_g_* = *R*_0_ is substituted into Formula (2), and *V_i_* = *V*_0_ = 0. In Figure 1, when ∆*R* = 0, the output of the bridge circuit voltage caused by |*R_g_* − *R*_0_| is *V_i_* ≠ 0. When ∆*R* ≠ 0, the output is *V_t_*. Then, the bridge voltage change caused by ∆*R* is *V*_0_ = *V_t_* − *V_i_*. Let *V_r_* = *V*_0_/*Er*; the voltage change caused by ∆*R* is:

(3) $$V_{0}=\left[\left(\frac{R_{g}+R_{f}}{R_{0}+R_{0}+2R_{f}}-\frac{1}{2}\right)-(\frac{R_{g}+R_{f}}{R_{0}+∆R+R_{0}+2R_{f}}-\frac{1}{2})\right]E$$

We substitute Formula (1) into Formula (3) to obtain a simplified Formula (4):

(4) $$\varepsilon =\frac{4V_{r}}{\kappa \left(1-2V_{r}\right)}(\frac{R_{0}+R_{g}}{R_{0}})$$

where *ε* is the accurate value of the three-wire strain calculation in Formula (4). The three-wire connection method can be used for static and dynamic strain measurements of a 1/4 bridge.

### 2.2. Spot-Welding Strain Sensor Packaging Solution

A high-performance adhesive was used to attach the resistance strain gauge to a thin steel sheet, and this assembly was then heat cured to create a spot-weldable strain sensor that increases the durability and service life of the strain gauge for use in long-term applications. The pasting, sealing, and maintenance of strain gauges render them unsuitable for cable production, and it is difficult to satisfy the construction schedule requirements of cables. However, spot-welded strain sensors can be prepared in the laboratory, and spot welding can be performed on the cable body. Thereafter, these strain sensors can be used. The thin steel sheet used in the test comprised stainless steel 304, the production standard was 06Cr19Ni10, the yield strength was 210 MPa, the tensile strength was 530 MPa, the modulus of elasticity was 2.1 × 10^5^ MPa, the shear modulus was 8.1 × 10^4^ MPa, and the Poisson’s ratio was 0.3. The strain gauge parameters used were as follows: 120 Ω.-1AA, length 7 mm, width 1.4 mm, both ends of the wire, and high-temperature resistance of 200 degrees.

**Figure 2:**
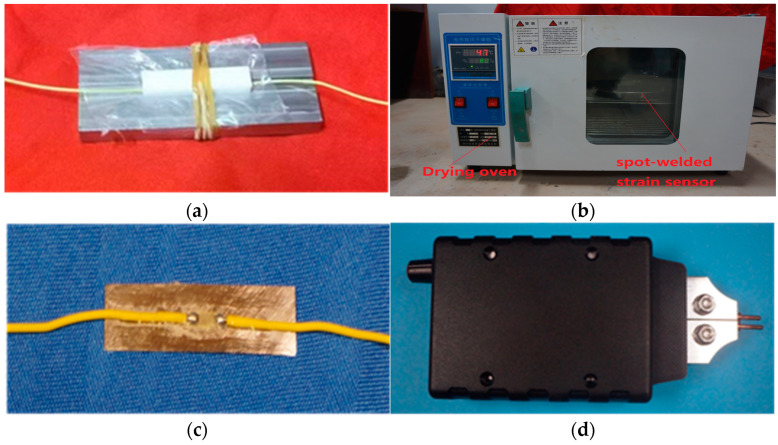
Craft tool and fabricated device. (**a**) Encapsulated resistance strain gauge device; (**b**) drying oven; (**c**) spot-welded strain sensor; (**d**) portable spot-welding machine.

In the present packaging of the resistance strain gauges, the pressure, temperature, and time are the three experimental factors affecting the curing. It is necessary to strictly ensure the standardization of this curing. Therefore, the typical production process is as follows: (1) The surface impurities of the thin steel sheet were removed, and sandpaper was used to polish out the cross stripes at an angle of 45° to the direction of the patch. The grinding surface was cleaned with an absorbent cotton ball soaked in acetone, and then scrubbed with absolute ethanol until there were no impurities on the cotton ball. (2) To ensure the accuracy of the pasting position of the strain gauge, a positioning line was drawn down the patch of the thin steel sheet and placed in the groove of the mold. (3) An appropriate quantity of adhesive was placed on the back of the resistance strain gauge and applied evenly with a brush; the strain gauge was placed on the thin steel sheet for positioning and pressed firmly for 30–60 s after covering the PTFE (polytetrafluoroethylene) film. Then, the PTFE groove plate and the rubber ring of the fixture device were used to apply pressure, as shown in Figure 2a. (4) To effectively eliminate the internal stress, the device was placed in a temperature box, the temperature was adjusted to approximately 30 °C, and the heat was maintained for 1–2 h for stabilization treatment, i.e., initial curing. The temperature was increased to 140 °C, maintained for 2 h, and subsequently decreased to room temperature (20–25 °C) to relieve the pressure. This temperature was then increased to 170 °C, maintained for 2 h, and subsequently decreased to room temperature, as shown in Figure 2b. The resulting spot-welded strain sensor is shown in Figure 2c.

In spot-welded strain sensors, strain measurements can be performed by welding the sensor to the measurement point on the steel strand using a special portable spot-welding machine (as shown in Figure 2d). Portable spot-welding machines offer the advantages of simple operation and fast welding speed; moreover, they obviate the need for a filler metal during welding. The current spot-welding process involved combining the workpiece lap and combination, applying pressure to the electrode, leveraging the resistance-based thermal effect generated based on the current flowing through the contact surface and its adjacent areas to heat the welding metal to a molten or plastic state, and then solidifying this metal to form an electrical connection [22,23]. This specific welding process for the strain sensor on the steel strand further included pressurizing the spot-welding strain sensor to maintain close contact at the appropriate position on the steel strand.

A portable spot-welding machine was subsequently used to select the appropriate current gear. The two electrodes were spot welded to the perimeter of the strain sensor, heat was generated at the contact point under the action of resistance and thermal effects, and a solder joint was formed after cooling. When the thin steel sheet and steel strand were joined using spot welding, the current output of the spot-welding machine was minimal, and the thin steel sheet was heated only at the solder joint position. The local heat generated at the solder joint during the welding process exhibited almost no damage to the steel strand and therefore did not damage the strain gauge.

## 3. Welding Strength and Strain Transmission of the Spot-Welded Strain Sensor

### 3.1. Weld Strength Test of the Metal Substrate

Three galvanized steel wires with a length of 400 mm and a diameter of 7 mm, and three thin steel sheets with dimensions of 26 mm × 7 mm × 0.05 m were selected for testing, and a ZQ-100 microcomputer tensile testing machine was used for tensile testing.

**Figure 3:**
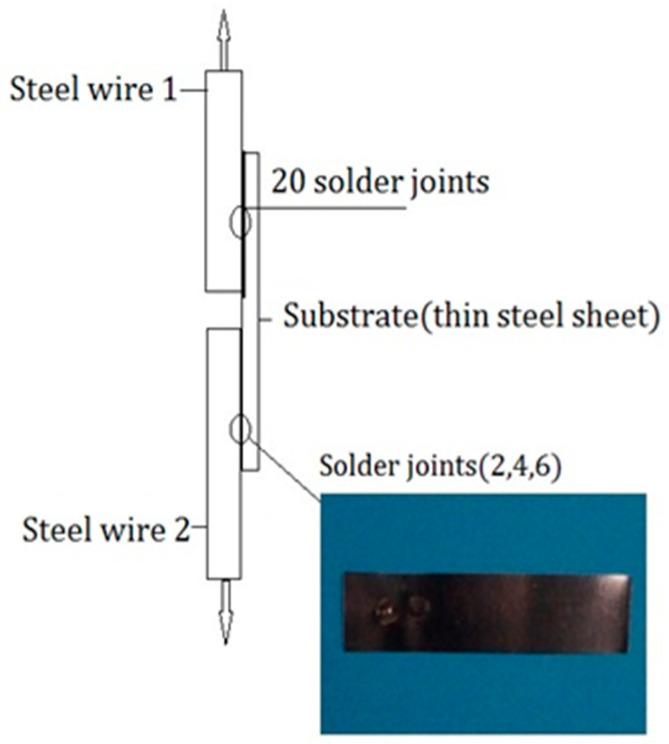
Schematic diagram of the welded joints of the steel sheet.

Three tensile tests were thus conducted. In each test, a steel wire was polished and scrubbed with pure ethanol, and a thin steel sheet was welded to the steel wire using spot welding. The corresponding solder joints are shown in Figure 3. When the thin steel sheet was desoldered, the tension of the steel wire was recorded, and the diameter of the welded joints was measured with a Vernier caliper to an average of 0.990 mm. Thus, the shear stress at the welded joints was derived and yielded an average value of 37.2 MPa, as shown in Table 1.

The test results reveal that the shear stress variance was minimal for the three pretensioned welded joints, and there was an average shear stress of 37.2 MPa, indicating reliable welding strength. These findings further indicate that the wire deformation through the welded joints can be stably transferred to the substrate, and this deformation can be increased by increasing the number of welded joints to increase the strength of the substrate and steel wire bonding, thereby increasing the operating range of the sensor used to measure the tension of the steel wire.

### 3.2. Theoretical Analysis of Sensor Strain Transfer

In contrast to the direct pasting of strain gauges, spot-welded strain sensors are packaged with thin steel sheets, and the recorded strain of the sensor deviates from the true strain of the component to be measured [24,25]. To analyze the strain transfer of spot-welded strain sensors, basic assumptions are made as follows: (1) the material is continuous, uniform, and isotropic, and the deformation process is linear elastic; (2) the matrix layer bears a uniform tensile strain, and the strain layer is deformed via the cementation layer; (3) the strain layer includes the same material as the matrix layer, including the same mechanical properties; (4) the strain gradient of each layer is the same, and the shear stress changes linearly with the thickness; (5) each layer exhibits only axial displacement in the direction of stretching; and (6) the cemented layer and the strain layer strain the matrix layer, thereby reducing the strain of the matrix layer.

**Figure 4:**
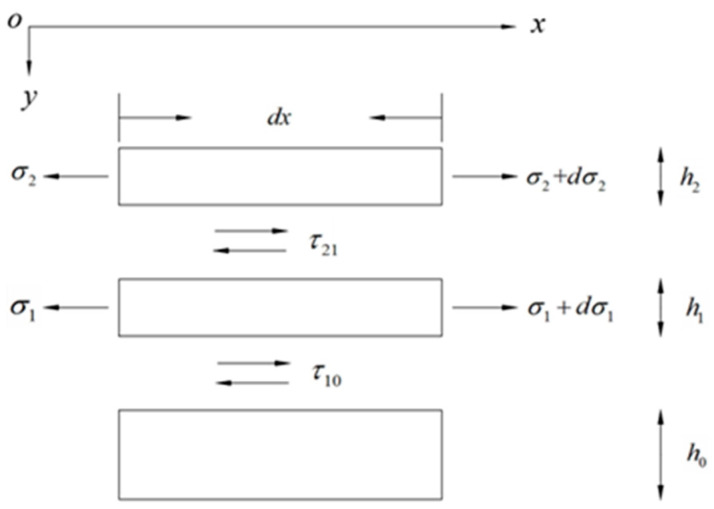
Force transfer diagram of each layer of the spot-welded strain sensor.

In Figure 4, *σ*_1_ and *σ*_2_ represent the positive stresses of the cemented layer and strain layer, respectively; *τ*_10_ and *τ*_21_ represent the shear stresses of the interface between the cemented layer and the matrix layer and between the strain layer and the cemented layer, respectively. *τ*_0_, *τ*_1_, and *τ*_2_ represent the shear stresses of the matrix, cementation, and strain layers, respectively. *ε*_0_, *ε*_1_, and *ε*_2_ represent the strain parameters of the matrix, cementation, and strain layers, respectively. *u*_0_, *u*_1_, and *u*_2_ represent the axial displacements of the matrix, cementation, and strain layers, respectively. *E*_0_, *E*_1_, and *E*_2_ represent the elastic moduli of the matrix, cementation, and strain layers, respectively. *G*_0_, *G*_1_, and *G*_2_ represent the shear moduli of the substrate, cementation, and strain layers, respectively. *h*_0_, *h*_1_, and *h*_2_ represent the thickness values of the substrate, cementation layer, and strain layer, respectively, and b is the width of the sensor.

In the case of force balance, the strain layer analysis is given as:

(5) $$d\sigma_{2}bh_{2}+\tau_{21}bdx=0$$

The cemented layer analysis is given as:

(6) $$d\sigma_{1}h_{1}-\tau_{21}bdx+\tau_{10}bdx=0$$

Because the strain gradient of each layer is the same, we obtain:

(7) $$\frac{d\varepsilon_{1}}{dx}=\frac{d\varepsilon_{2}}{dx}$$

The relationship between the stress and strain of each layer is obtained as:

(8) $$\frac{d\sigma_{n}}{dx}=E_{n}\frac{d\varepsilon_{n}}{dx}$$

It can be obtained from Equation (2) and Equations (5) to (7) that:

(9) $$\tau_{21}=-E_{2}h_{2}\frac{d\varepsilon_{2}}{dx}\;$$

(10) $$\tau_{10}=-\left(E_{2}h_{2}+E_{1}h_{1}\right)\frac{d\varepsilon_{2}}{dx}$$

Given that the shear stress of each layer varies linearly with thickness, for the cementitious layer, we obtain:

(11) $$\tau_{1}=\frac{\tau_{10}-\tau_{21}}{h_{2}}\left(y-h_{2}\right)+\tau_{21}\;(h_{2}<y<h_{2}+h_{1})$$

Using expression $\tau_{1}=G_{1}\frac{du}{dy}$ , we simultaneously integrate both sides of Equation (11) with respect to *y* to obtain:

(12) $$\int_{h_{2}}^{h_{2}+h_{1}}G_{1}\frac{du}{dy}dy=\int_{h_{2}}^{h_{2}+h_{1}}\left[\frac{\tau_{10}-\tau_{21}}{h_{1}}\left(y-h_{2}\right)+\tau_{21}\right]dy$$

Simplifying Equation (12) gives:

(13) $$G_{1}\left(u_{1}-u_{2}\right)=-\frac{1}{2}h_{1}\left(2E_{2}h_{2}+E_{1}h_{1}\right)\frac{d\varepsilon_{2}}{dx}$$

By deriving both sides of Equation (13) with respect to *x* and simplifying it, the following is obtained:

(14) $$\varepsilon_{2}=\varepsilon_{1}+\frac{1}{2}\frac{h_{1}}{G_{1}}\left(2E_{2}h_{2}+E_{1}h_{1}\right)\frac{d^{2}\varepsilon_{2}}{dx^{2}}\;$$

The base layer is described as:

(15) $$\tau_{0}=\frac{\tau_{10}}{h_{0}}\left(h_{2}+h_{1}+h_{0}-y\right)\;(h_{2}+h_{1}<y<h_{2}+h_{1}+h_{0})$$

Using the expression $\tau_{0}=G_{0}\frac{du}{dy}$ and simultaneously integrating both sides of Equation (15) with respect to *y* obtains:

(16) $$\int_{h_{2}+h_{1}}^{h_{2}+h_{1}+h_{0}}G_{0}\frac{du}{dy}dy=\int_{h_{2}+h_{1}}^{h_{2}+h_{1}+h_{0}}\frac{\tau_{10}}{h_{0}}\left(h_{2}+h_{1}+h_{0}-y\right)dy$$

Simplifying Equation (16) gives:

(17) $$G_{0}\left(u_{0}-u_{1}\right)=-\frac{1}{2}h_{0}\left(E_{2}h_{2}+E_{1}h_{1}\right)\frac{d\varepsilon_{2}}{dx}$$

By deriving both sides of Equation (17) with respect to *x* and simplifying it, the following is obtained:

(18) $$\varepsilon_{1}=\varepsilon_{0}+\frac{1}{2}\frac{h_{0}}{G_{0}}\left(E_{2}h_{2}+E_{1}h_{1}\right)\frac{d^{2}\varepsilon_{2}}{dx^{2}}$$

From Equations (14) and (18), the following is obtained:

(19) $$\varepsilon_{2}=\varepsilon_{0}+\frac{1}{2}\left[\frac{h_{0}}{G_{0}}\left(E_{2}h_{2}+E_{1}h_{1}\right)+\frac{h_{1}}{G_{1}}(2E_{2}h_{2}+E_{1}h_{1})\right]\frac{d^{2}\varepsilon_{2}}{dx^{2}}$$

Thus, the following is obtained:

(20) $$\frac{1}{K^{2}}=\frac{1}{2}\left[\frac{h_{0}}{G_{0}}\left(E_{2}h_{2}+E_{1}h_{1}\right)+\frac{h_{1}}{G_{1}}(2E_{2}h_{2}+E_{1}h_{1})\right]$$

Substituting Equation (20) into Equation (19) yields:

(21) $$\frac{d^{2}\varepsilon_{2}\left(x\right)}{dx^{2}}-k^{2}\varepsilon_{2}\left(x\right)=-k^{2}\varepsilon_{0}$$

Solving Equation (21) results in:

(22) $$\varepsilon_{2}\left(x\right)=C_{1}e^{kx}+C_{2}e^{-kx}+\varepsilon_{0}$$

According to the boundary conditions: $\varepsilon_{2}\left(l\right)=\varepsilon_{2}\left(-l\right)=0$ :

(23) $$C_{1}=C_{2}=-\frac{\varepsilon_{0}}{2\cos h(kl)}$$

Substituting Equation (23) into Equation (22) yields:

(24) $$\varepsilon_{2}\left(x\right)=\left[1-\frac{\cos h(kx)}{\cos h(kl)}\right]\varepsilon_{0}$$

The average strain of the entire strain layer is thus obtained as:

(25) $$\bar{\varepsilon_{2}}=\frac{\varepsilon_{0}}{2l}\int_{-l}^{l}\left[1-\frac{\cos h(kx)}{\cos h(kl)}\right]dx=\left[1-\frac{\sin h(kl)}{kl\cos h(kl)}\right]\varepsilon_{0}$$

Thus, the average strain transfer coefficient of the entire strain layer is given as:

(26) $$\alpha =\bar{\frac{\varepsilon_{2}}{\varepsilon_{0}}}=1-\frac{\sin h(kl)}{kl\cos h(kl)}$$

The strain transfer coefficient of the spot-welded strain sensor is related to the thickness of the base layer *h*_0_, the thickness of the cemented layer *h*_1_, the thickness of the strained layer *h*_2_, the elastic modulus *E*_1_ of the cemented layer, the elastic modulus *E*_2_ of the strained layer, the shear modulus *G*_0_ of the cemented layer, the shear modulus *G*_1_ of the cemented layer, and the bonding length of the strained layer. For other sensors packaged with substrates, the theoretical derivation process is similar, and the strain transfer equation has the same form; however, the expression for *k* is different, reflecting the different laws that influence various strain transfers.

### 3.3. Finite Element Simulation of Sensor Strain Transfer

To further study the strain transfer of spot-welded strain sensors, a finite element method was used. The finite element analysis was implemented in ABAQUS to describe the strain transfer law of the sensor in the elastic stage. In this model, each component was regarded as an ideal linear elastic material, regardless of plastic deformation. The geometric and mechanical parameters of each sensor component are listed in Table 2.

**Figure 5:**
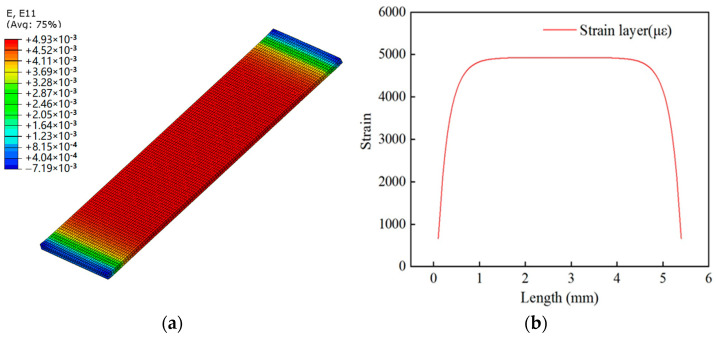
Finite element model and strain curve. (**a**) Strain program of the strained layer simulated using the finite element method; (**b**) strain curve along the length of the strained layer.

A finite element model of the spot-welded strain sensor was thus established, where each part of the sensor was assigned the solid element C3D8R. The packaging substrate was fixed to the steel plate specimen via spot welding, and the contact surface between the two was restrained using ties. In addition, the contact surfaces between the packaging substrate and the bonding layer and between the bonding layer and the strain layer were constrained using ties. One end of the steel plate specimen was fixed, and a displacement load of one unit was applied to the other end. A tie constraint was used in the finite element analysis, and the finite element mesh size was 0.05 mm. The substrate is fixed on the specimen by means of resistance spot welding. Since there is no relative slip between the interface of the two (there is no dewelding during the stretching process), spot welding is reflected in the model as the tie constraint between the substrate and the specimen. The strain cloud diagram obtained after solving and analyzing the finite element model is shown in Figure 5a. The model was postprocessed to extract the strain value of the strain layer along its length; the results are shown in Figure 5b.

Within a small range, the strains at both ends of the strain layer were lower than those at the steel plate specimen, whereas the strain in the middle area of the strain layer was relatively uniform and close to that of the steel plate specimen. The resistance strain gauge facilitates the measurement of the average strain of the sensitivity grid region; therefore, the strain value of the component is averaged to obtain the average strain $\bar{\varepsilon }$ ≈ 4.884 × 10^−3^ and the strain of the steel plate specimen *ε* ≈ 5 × 10^−3^. Therefore, the strain transfer efficiency of the finite element simulation can be obtained as follows:

(27) $$\eta =\frac{\bar{\varepsilon }}{\varepsilon }\approx \frac{4.884\times 10^{-3}}{5\times 10^{-3}}\approx 97.68\%\;$$

Based on the theoretical derivation of the equation, the strain transfer efficiency was obtained by substituting the parameters from Table 2 as follows:

(28) $$\alpha =1-\frac{\sin h\left(kl\right)}{kl\cos h\left(kl\right)}\approx 96.97\%$$

## 4. Characterization of a Spot-Welded, Strain Sensor-Equipped Smart Cable

The spot-welded strain sensor was fixed on the steel strand, a configuration that is referred to as a spot-welded, strain sensor-equipped intelligent strand cable. Accordingly, the performance of this system is evaluated. The strain sensing, temperature sensitivity, stress relaxation, and other related tests of the spot-welded, strain sensor-equipped intelligent strand cable were conducted in the laboratory.

### 4.1. Performance Testing of Smart Steel Strand Strain Sensing

To investigate the strain-sensing performance of the spot-welded, strain-sensor-equipped intelligent steel strands, a static loading test was performed. The test material was Φ15.2 galvanized steel stranded wire with a tensile strength of 1860 MPa. The test equipment included a tension oil pump, hydraulic jack, reaction frame, pressure sensor, standard load-measuring instrument, wireless strain collector, clip anchor, and anchor plate. The resistive strain gauge model used in the test was a BMB120-1AA-the P300, with the grid material being a copper–nickel alloy.

The basic testing process was as follows: (1) Polishing was employed to remove the galvanized layer on the surface of the steel strand, followed by cleaning with alcohol. (2) The strain sensor was welded to the outer wire of the steel strand using a spot-welding machine. In this process, a one-handed pressure welding strain gauge and one-handed welding machine collision welding were used. The number and sequence of the welding points, as well as the spacing distance between the welding points, should generally be controlled at 0.8 mm. The welding points cross each other, with the first point traversing the strain gauge’s axial length center point, down the lead line direction of the welding. The second connection is welded at the midpoint on the other side of the strain gauge. The third step is to weld upward along the opposite side (i.e., the first-step welding side). The fourth step is to take the starting point of the second step and weld upward until the end of the cable. (3) Steel strand testing was performed. The loading utilized graded tension, and the load class was divided into six levels: 10 kN, 30 kN, 60 kN, 90 kN, 120 kN, and 150 kN in ascending order. The strain values need to be collected for pretensioning three times, cyclic loading three times, and for each stage of loading. The strain-sensing performance test was performed in an RHPW-24CT walk-in, constant temperature and humidity chamber of the laboratory at room temperature (20 °C–25 °C).

**Figure 6:**
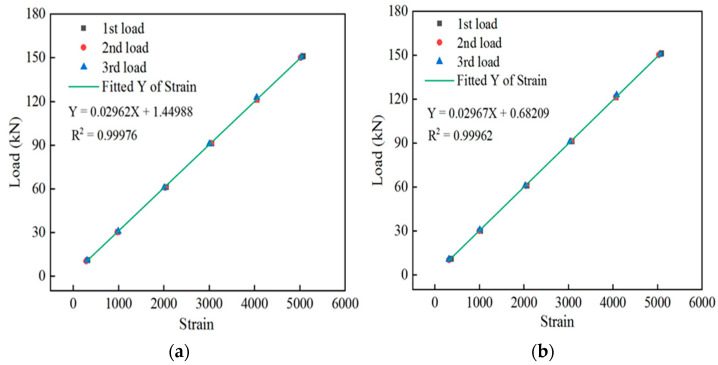
Relationship between the load and strain of the sensor: (**a**) relationship between the load and strain of sensor #1 and (**b**) relationship between the load and strain of sensor #2.

A DH3819 wireless strain collector was used to collect data from the spot-welded strain sensor. Three cyclic loadings were applied to the spot-welded, strain sensor-equipped smart steel strands. These three strain readings were obtained for each level of the tension load, and the resulting average strain value was obtained. The fitting relationship curves of the load and strain of sensor #1 and sensor #2 are shown in Figure 6a,b, respectively.

By substituting the collected sensor strain value into the calibration equation, the corresponding tension value was obtained and compared with that which was obtained by the pressure sensor, and the measurement error of the spot-welded strain sensor was therefore obtained. The tested tension and error values for sensor #1 and sensor #2 are listed in Table 3.

Table 3 shows that under various tension loads, the tension errors of sensor #1 and sensor #2 are within 1.26%, and the accuracy of the recordings is high. The performance of the sensors was repeatable across different loading times. This result demonstrates that the spot-welded, strain sensor-equipped intelligent steel strand can accurately sense its tension, monitor the entire process of the steel strand under load, and exhibit favorable repeatability.

### 4.2. Temperature Sensitivity Performance Test and Compensation of Intelligent Steel Strands

Loading tests were further performed at different temperatures to explore the temperature sensitivity of the spot-welded, strain sensor-equipped intelligent steel strands. The test materials and equipment used were identical to those used for the strain-sensing performance tests. Because it was necessary to adjust the temperature during the test, the RHPW-24CT walk-in, constant temperature and humidity chamber were temperature controlled.

The testing steps were as follows: (1) The surface treatment of the steel strand and the welding of the spot-welded strain sensor were the same as those used in the strain sensing performance test, and a steel strand-welded sensor was placed statically next to the reaction frame for temperature compensation. (2) The control temperature was increased from 0 °C to 40 °C in increments of 10 °C. The temperature at each stage was held constant for 2 h, and pretensioning was conducted three times before the test. (3) When the intelligent strand test was conducted at each stage temperature, the loading mechanism and strain acquisition were the same as those used for the strain-sensing performance test.

**Figure 7:**
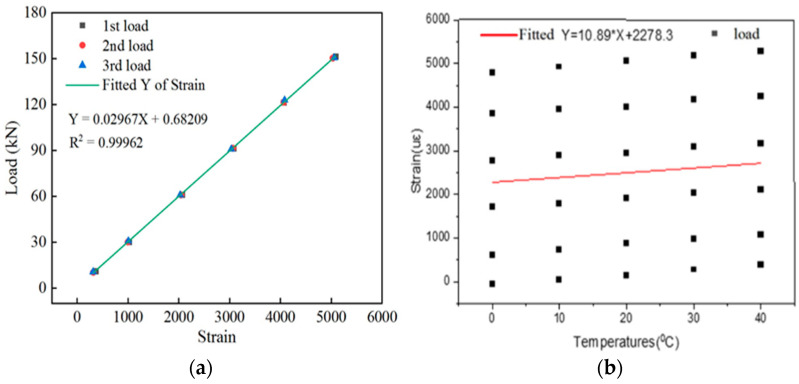
(**a**) Relationship between the load and strain at different temperatures; (**b**) Relationship between the strain and temperature under different loads.

The sensor strain data corresponding to the tension loads across all levels were collected, as listed in Table 4. The trend of the sensor load with applied strain at different temperatures was plotted, as shown in Figure 7a. The influence of temperature on the test manifested as a uniform shift in the curve between the tensile load and the sensor strain at different temperatures. The higher the test temperature under the same tensile load was, the greater the test strain value was. A temperature compensation treatment of the spot-welded strain sensor was performed as follows:

(29) $$y_{t}\left(x\right)=A+B(x-\lambda \left(T-T_{0}\right))$$

where *A* and *B* are the equation parameters, *λ* is the temperature compensation coefficient, *T* is the actual temperature, and *T*_0_ is the standard temperature. With Formula (29), three variables, namely, the force, temperature, and strain, are calculated by using the least squares method of setting the force, and the transverse temperature and longitudinal strain are used to calculate the two variables, namely, the temperature and longitudinal strain. Then, a scatterplot of the strain increase at each position of the welded strain gauge at different temperatures is drawn, and the linear fitting curve is shown in Figure 7b.

(30) y=2278.3+10.89×x

Therefore, Formula (30) represents the slope when the temperature compensation coefficient λ is 10.89. The temperature sensitivity coefficient of the meter is represented by the slope of the fitted curve. Equation (29) compensates for the difference when the temperature compensation equation is $x=x_{c}-\lambda (T_{c}-T_{0})$ . The value of $x_{c}$ is a measurement value. The values of $T_{c}$ are the measured temperature values. The $T_{0}$ values are the standard temperature values. This analysis shows that the linear fitting equation at various temperatures is *y_t_*(*x*) = 0.68209 + 0.02967 × (*x* − 10.89 × (*T* − *T*_0_)), where the standard temperature *T*_0_ is 20 °C. The sensor strain values collected at different temperatures were substituted into the calibration equation after temperature compensation, after which the corresponding tension values were obtained and subsequently compared to the values obtained using the pressure sensor. The test error of the spot-welded strain sensor was obtained for the tension of the steel strand. The tension values measured by the sensors at different temperatures and their errors are presented in Table 4.

Under different temperatures and tensile loads, the full capacity error of the temperature–tension–force detection based on the spot-welded strain sensor was 5.0%. Moreover, the tension test’s compensation full capacity error was within 1.0%, and the accuracy of the compensation results was high, indicating that the substrate strain sensor-equipped intelligent steel strand can accurately sense its tension at different temperatures and has favorable repeatability.

### 4.3. Stress Relaxation Performance Test of the Intelligent Steel Strands

High-strength steel strands are typically used in high-stress states. If the total strain is constant, then the stress decreases with time. In such conditions, stress relaxation is a concern. To explore the stress relaxation performance of the substrate-type strain smart steel strand, the strain-sensing performance of the spot-welded strain sensor was tested before and after the relaxation of the steel strand. A stress relaxation test was conducted in the relaxation laboratory. The test was performed according to the specifications GB/T 5224-2014 “Steel Strand for Prestressed Concrete” [26], GB/T 21839-2019 “Test Method for Steel for Prestressed Concrete” [27], and GB/T 10120-2013 “Test Method for Tensile Stress Relaxation of Metal Materials” [28]. The test material was a Φ15.2 galvanized steel strand with a tensile strength of 1860 MPa, and the test equipment was a KRDW-SC300 computer-controlled steel strand relaxation tester.

**Figure 8:**
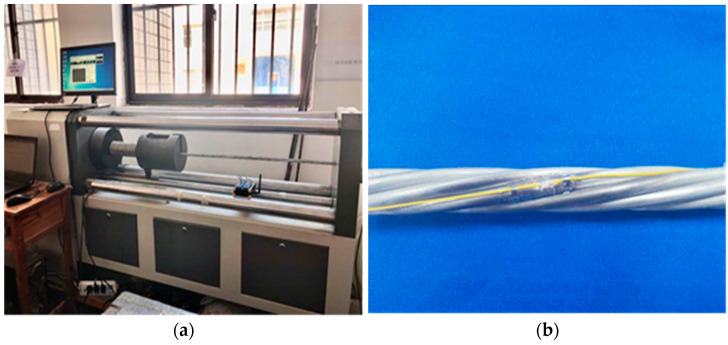
Relaxation test and steel strand. (**a**) Relaxation test of intelligent galvanization; (**b**) sensor welded to the steel strand.

The basic steps of the test were as follows: (1) The indoor temperature of the relaxation test room was maintained at approximately 20 °C, and the steel strand was kept at a constant temperature for 24 h. (2) The steel strand was placed on the relaxation testing machine, both ends of the steel strand were clamped with a fixture and kept straight, and two strain sensors were welded on the steel strand. (3) The stress sensing test was performed before the relaxation test. This loading utilized graded tension, and the corresponding load was divided into 6 levels: 10 kN, 30 kN, 60 kN, 90 kN, 120 kN, and 150 kN. Pretensioning was applied three times, and the load test was performed once. Each loading stage was performed three times. (4) Loading was performed up to 180 kN at a uniform rate, the loading rate was 220 MPa/min, the force was applied smoothly without oscillation, and the loading was completed within 6 min. The holding time was 2 min, and the relaxation test was subsequently performed (5) until the end of the 120 h test; (6) the stress sensing test was conducted after the relaxation test. The same test environment and loading mechanism were used during the various stress sensing tests as occurred before the relaxation test. The stress relaxation performance test was recorded in a relaxation test chamber, as shown in Figure 8a,b.

**Figure 9:**
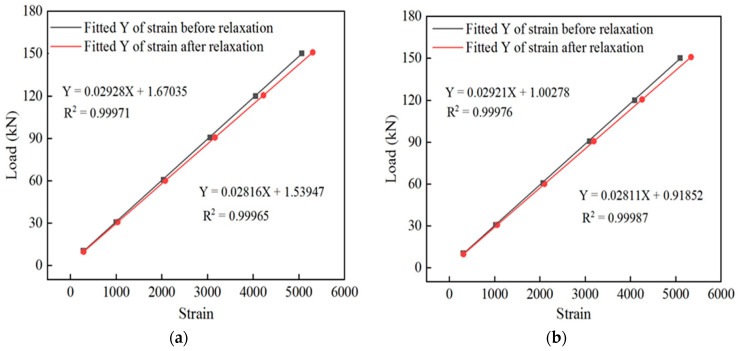
Relationship between load and strain. (**a**) Relationship between the load and strain before and after relaxation of sensor #1; (**b**) relationship between the load and strain before and after relaxation of sensor #2.

**Figure 10:**
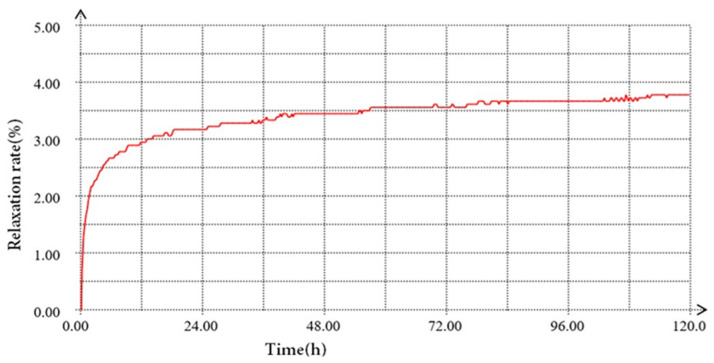
The relaxation rate curves of intelligent galvanized steel strands over time.

The sensor strain data corresponding to the tension load at each stage were collected separately. The strain-to-load curves before and after relaxation of sensor #1 and sensor #2 are shown in Figure 9a,b, respectively, and the relaxation rate of the intelligent galvanized steel strands with time is shown in Figure 10.

The relaxation rate of the spot-welded, strain intelligent galvanized steel strand relaxation test was 3.78% after 120 h. The analysis showed that the strain test performance of the spot-welded strain sensor was relatively stable before and after the relaxation test. Compared with that before relaxation, the sensitivity of sensor #1 after relaxation increased by 3.99%, and the sensitivity of sensor #2 increased by 3.96%, with an increasing mean value of 3.97%, which are essentially equal to the relaxation rate of spot-welded, strain intelligent galvanized steel strands. The linear fits of sensor #1 and sensor #2 before and after relaxation are greater than 0.999, and a favorable linear relationship remained, between the strain value collected during the test, and the tension load value collected after relaxation. This result demonstrates that the relaxation of the galvanized steel strand will affect the test results, but the magnitude of the influence is basically the same as that of the relaxation rate. The spot-welded, strain smart galvanized steel strand exhibits better-sensing linearity before and after relaxation.

The strain test data before and after the relaxation of the intelligent galvanized steel strand are used to obtain the corresponding tension value, and the tension test error is obtained. The tension values and their errors before and after the relaxation of sensor #1 and sensor #2 are shown in Table 5. Table 5 shows that under tension loading at all levels before and after relaxation, the tension test errors of sensor #1 and sensor #2 are within 2.45%, indicating the high accuracy of sensing tension in spot-welded, strain-sensor-equipped intelligent galvanized steel strands before and after relaxation. The relaxation rate of the smart steel strand cable after 120 h was 3.78%, with an influence on the sensor accuracy error of 3.97%.

### 4.4. Performance Test of the Intelligent Steel Strand Cable

**Figure 11:**
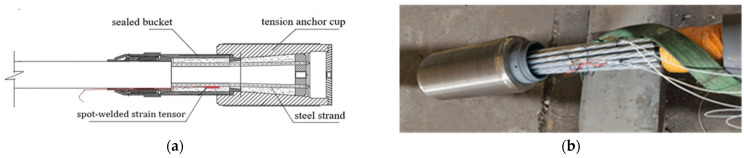
Smart steel strand cables: (**a**) schematic diagram of the anchor structure and sensor location; (**b**) photograph of the anchorage after sensor installation.

The cable body of the steel strand cable used in the test comprised 19 high-strength, seven-wire steel strands, and the two ends of the cable body were consolidated with anchorage by extrusion. The gap in the anchor cup was filled with a mixture of special epoxy resin, zinc powder, and cast steel. After curing at 180 °C, the steel strands were combined with the steel strands, and the cable body was wrapped with an HDPE (high-density polyethylene) sheath to protect it from environmental erosion and achieve effective protection. This cold-cast anchorage steel strand cable not only resisted high stress but also exhibited excellent fatigue resistance, as shown in Figure 11.

To explore the strained relationship between intelligent steel strands and cables, intelligent cables with parallel steel strands were fabricated, and static loading tests were performed on them. The cable body of the parallel-strand intelligent cable was composed of 19 galvanized steel strands with a tensile strength of 1860 MPa and a diameter of 15.24 mm. The nominal breaking cable force *P*_b_ was 4940 kN, and the design cable force was 0.4 *P*_b_ = 1976 kN. Two welded strain sensors and a temperature sensor were built into the galvanized steel strand at the anchor site, and the sensor wires were routed and protected using extension cylinders.

These tests were performed on a 1200 T static load test bench (China, Liuzhou Liuzhou OVM Machinery Co., Ltd.). A hydraulic oil pump produced by the OVM and a YCW1200 jack were used as test instruments. The standard pressure sensor was a CL-YB-12MN anchoring force sensor with a maximum measuring range of 12,000 kN and an uncertainty of 0.02. The temperature sensor model was PT100, and the A-level precision was 0.1. The Donghua static strain test and analysis system was used for the strain measurement.

**Figure 12:**
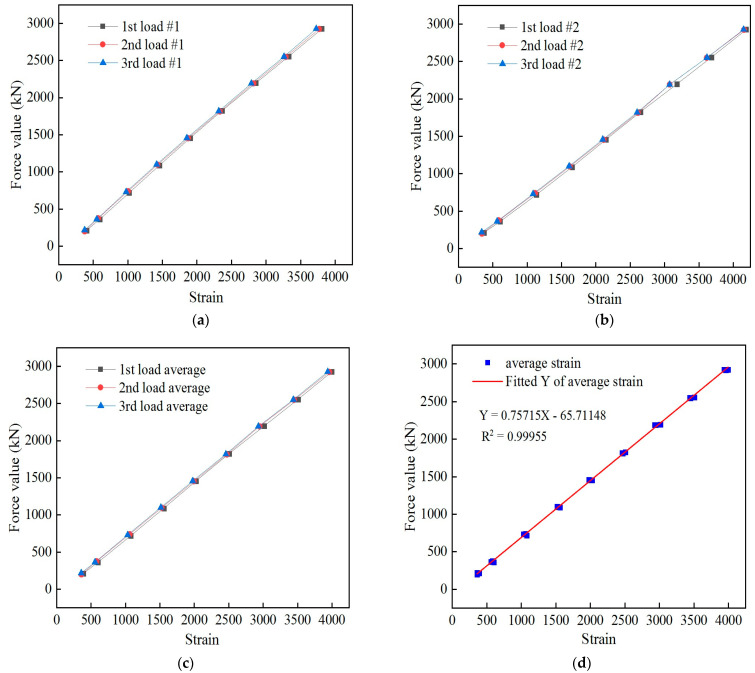
Relationship between the force of the smart cable and the sensor strain. (**a**) Relationship between the force on the smart cable and the strain in sensor #1; (**b**) relationship between the force on the smart cable and the strain in sensor #2; (**c**) intelligent cable force vs. average strain; (**d**) fitting curve of the force on the smart cable and average strain.

At the beginning of these tests, the cable was pretensioned three times. The loading system of the static load test included loading that started from zero to 0.6*P*_b_, with levels of loading separated by 0.06*P*_b_. The corresponding strain data were recorded after the load was held for 5 min, and the loading and unloading were repeated three times. After the test, the data were processed and analyzed, and the relationships between the force and strain of sensor #1 and sensor #2 were obtained after loading three times, as shown in Figure 12a,b. The force and average strain of the sensor are shown in Figure 12c, and the relationship between the force and average strain is shown in Figure 12d.

The linear fitting equation between the force of the intelligent cable and the average strain of the spot-welded strain sensor is *y* = −65.71148 + 0.75715*x*. There was a strong linear relationship between the force measured by the anchoring force sensor and the strain measured by the spot-welded strain sensor, and these results were repeatable. This outcome demonstrates that the strain transmissions of the welded strain sensor, welded strain sensor intelligent steel strand, and welded strain intelligent cable are consistent and exhibits an ideal strain synergy. The proposed intelligent spot-welded strain cable can therefore satisfy the requirements of cable force monitoring.

To further evaluate the accuracy and reliability of the cable force of the intelligent cable static load test, the cable force obtained using the sensor fitting formula was compared with that measured by the anchor force sensor. The corresponding results are summarized in Table 6, which shows that the full-scale error analysis was conducted by comparing the self-perceived cable force value of the spot-welded, strain sensor-equipped intelligent steel strand cable with the cable force value tested using the anchor force sensor. The maximum F.S. (full scale) error of the cable force of the intelligent steel strand cable was determined to be 1.3%, indicating that the smart steel strand cable exhibited high precision and reliability. Based on a spot-welded strain sensor that was placed within a cable extension tube, a new type of intelligent steel strand cable was developed. This intelligent cable can be directly transported to construction sites for cable hoisting and utilized for practical applications after laboratory calibration.

## 5. Conclusions

To overcome problems such as the low survival rate of strain sensors during cable installation, the adhesive easily falls off after working for a period of time and cannot be monitored for a long time. Corresponding principles and packaging for spot-welded strain sensors were introduced, wherein the welding strength and strain transfer theory were applied. Experimental studies were thus conducted to characterize the developed spot-welded, strain sensor-equipped intelligent steel strands and intelligent cables in this work. The main conclusions are as follows:

(1) A thin steel sheet was welded onto a galvanized steel wire with a diameter of 7 mm, and the tensile test results showed that the average shear stress of the thin steel sheet and galvanized steel wire weld was 37.2 MPa.
(2) The strain transfer theory of a spot-welded strain sensor was applied to the component to be measured, and a finite element model of the sensor was established. The analysis showed that the strain transfer efficiency of the spot-welded strain sensor was greater than 96%, and the strain loss was small.
(3) Performance tests of the spot-welded, strain sensor-equipped intelligent steel strand demonstrated that its train-sensing performance was superior, and the combination of the spot-welded strain sensor and steel strand had favorable adaptability and transmissibility. Static load tests of the spot-welded, strain sensor-equipped intelligent steel strand cable demonstrated that the spot-welded strain sensor was functional in the cable. The maximum cable force deviation was only −1.38%, the test accuracy was high, and the sensing linearity and repeatability were favorable.
(4) Under various temperatures and tensile loads, the set force, transverse temperature, and longitudinal strain coordinate methods are proposed, and linear fitting is adopted via the least squares method. The slope of the line is a coefficient, and the calibrated force value formula uses the coefficient for temperature compensation. After considering temperature compensation, the tension test of the spot-welded strain sensor compensates for the full capacity error within 1.0%. The intelligent strand exhibited favorable sensing linearity, and its tension-sensing accuracy was high.
(5) The relaxation rate of the smart steel strand cable after 120 h was 3.78%, with an influence on the sensor accuracy error of 3.97%. The proposed spot-welded, strain-sensor-equipped smart steel strand cable can be applied to the long-term tension monitoring of cable-supported structures in various architectural settings, such as bridges and stadiums, from their construction to operation.
(6) The proposed substrate-type sensor is mounted by spot welding. A detailed study of the stability and reliability of the measurements after mounting has been conducted, but data from long-term monitoring application tests in actual engineering are lacking. Our next step will be to carry out a long-term monitoring experimental study, and the long-term monitoring experimental data will be compared and analyzed.

## Figures and Tables

**Table 1:** Welding strength test results for thin steel sheets and steel wires.

Test	Number of Solder Joints/n	Tension/N	Shear Stress/MPa
1	2	54.7	35.5
2	4	118.2	38.4
3	6	174.2	37.7
Average	/	/	37.2

**Table 2:** Finite element model parameters of the spot-welded strain sensor.

Part	Geometry/mm	Elastic Modulus/MPa	Poisson’s Ratio	Shear Modulus/MPa
Strain layer	5.5 × 1.8 × 0.1	1.1 × 10^5^	0.33	4.1 × 10^4^
Cement layer	5.5 × 1.8 × 0.1	1.0 × 10^3^	0.35	3.7 × 10^2^
Packaging substrate	26 × 7 × 0.05	2.1 × 10^5^	0.3	8.1 × 10^4^
Steel plate test piece	200 × 50 × 5	2.1 × 10^5^	0.3	8.1 × 10^4^

**Table 3:** Tension and error values of the intelligent steel strand.

Number of Loads (*N*)	The Pressure Sensor Indicator (*F*)/kN	Tension Values Tested by Sensor #1 (*T*_1_)/kN	Error Value of Sensor #1 (*ε*_1_)/%	Tension Values Tested by Sensor #1 (*T*_2_)/kN	Error Value of Sensor #2 (*ε*_2_)/%	Average Tension Value (*T*)/kN	Average Error Value (*ε*)/%
1	30.41	30.40	−0.02	30.80	1.28	30.60	0.63
	61.23	62.12	1.46	61.89	1.07	62.00	1.26
	91.39	91.86	0.51	91.89	0.54	91.87	0.53
	121.32	121.31	−0.01	121.16	−0.13	121.23	−0.07
	151.39	151.52	0.09	151.63	0.16	151.58	0.12
2	30.20	30.32	0.41	30.29	0.30	30.31	0.36
	60.89	61.57	1.11	61.38	0.80	61.47	0.96
	91.33	91.66	0.36	91.58	0.27	91.62	0.32
	121.41	121.55	0.11	121.17	−0.19	121.36	−0.04
	150.21	150.06	−0.10	149.92	−0.19	149.99	−0.15
3	30.85	30.83	−0.06	30.55	−0.98	30.69	−0.52
	60.78	60.95	0.29	60.81	0.05	60.88	0.17
	90.83	90.46	−0.41	90.66	−0.18	90.56	−0.29
	122.77	121.39	−1.12	121.76	−0.82	121.58	−0.97
	150.36	150.49	0.09	150.74	0.25	150.62	0.17

Note: In the table, *N* is the number of loads, *F* is the reading of the load measuring instrument, *T*_1_ and *T*_2_ are the tension values recorded by sensor #1 and sensor #2, respectively, *ε*_1_ and *ε*_2_ are the corresponding error values, *T* is the average tension value, and *ε* is the average error value.

**Table 4:** Strain values and forces of intelligent galvanized steel strands at different temperatures.

Temperature /°C	Test Force Value/kN	Strain Value /με	Temperature Computational Force/kN	Compensation Computational Force/kN	Compensation Full Capacity Error (ε)/%
0	11	−60	4.7	10.9	−0.1
	31	613	23.9	30.1	−0.6
	61	1709	55.2	61.5	0.3
	91	2766	85.5	91.7	0.5
	121	3841	116.2	122.5	1.0
	151	4792	143.4	149.7	−0.8
10	11	51	7.8	11.0	0
	31	736	27.4	30.5	−0.3
	61	1787	57.5	60.6	−0.3
	91	2899	89.3	92.4	0.9
	121	3940	119.1	122.2	0.8
	151	4921	147.1	150.2	−0.5
20	11	138	10.3	10.3	−0.5
	31	885	31.7	31.7	0.5
	61	1918	61.2	61.2	0.1
	91	2948	90.7	90.7	−0.2
	121	3999	120.7	120.8	−0.1
	151	5059	151.1	151.1	0.1
30	11	273	14.2	11.1	0.1
	31	968	34.1	31.0	0
	61	2033	64.5	61.4	0.3
	91	3085	94.6	91.5	0.3
	121	4166	125.5	122.4	0.9
	151	5171	154.3	151.2	0.1
40	11	392	17.6	11.4	0.3
	31	1070	37.0	30.8	−0.1
	61	2111	66.8	60.6	−0.3
	91	3167	97.0	90.8	−0.1
	121	4241	127.7	121.5	0.3
	151	5267	157.0	150.8	−0.1

**Table 5:** Tension and error values of the intelligent galvanized steel strands before and after relaxation.

Relaxation	The Pressure Sensor Indicator (*F*)/kN	Tension Values Tested Using Sensor #1 (*T*_1_) /kN	Error Value of Sensor #1 (*ε*_1_)/%	Tension Values Tested Using Sensor #1 (*T*_2_)/kN	Error Value of Sensor #2 (*ε*_2_)/%	Average Tension Value (*T*)/kN	Average Error Value (*ε*)/%
Before	30.9	30.61	−0.95	30.83	−0.22	30.72	−0.58
	60.7	61.23	0.88	61.26	0.92	61.25	0.90
	90.6	91.10	0.55	91.19	0.66	91.15	0.60
	120.2	120.33	0.10	120.39	0.16	120.36	0.13
	150.3	149.72	−0.38	149.62	−0.45	149.67	−0.42
After	30.7	31.39	2.25	30.85	0.50	31.12	1.38
	60.0	60.62	1.04	60.60	1.00	60.61	1.02
	90.6	88.38	−2.45	89.15	−1.60	88.76	−2.03
	120.5	121.36	0.72	121.43	0.77	121.40	0.74
	150.9	151.17	0.18	150.77	−0.09	150.97	0.05

**Table 6:** Cable force and error from the parallel-strand intelligent cable static load test.

Standard Load/kN	Strain in Sensor #1/με	Strain in Sensor #2/με	Average Strain/με	Cable Force/kN	Error/%F.S. (Full Scale)
213.3	404.7	373.5	389.1	228.8	−0.5
363.1	594.8	608.1	601.4	389.6	−0.9
719.7	1019.9	1133.5	1076.7	749.5	−1.0
1089.9	1459.8	1655.5	1557.6	1113.6	−0.8
1456.1	1902.0	2148	2025.0	1467.5	−0.4
1826.1	2364.5	2648.2	2506.3	1832.0	−0.2
2196.4	2852.4	3177.1	3014.7	2217.0	−0.7
2555.8	3329.7	3681.4	3505.5	2588.6	−1.1
2925.1	3806.0	4187.2	3996.6	2960.5	−1.2
200.1	377.6	342.5	360.0	206.8	−0.2
376.2	572.6	588.6	580.6	373.8	0.1
740.2	1005.3	1118.5	1061.9	738.2	0.1
1097.1	1431.0	1623.2	1527.1	1090.5	0.2
1453.1	1872.9	2116.3	1994.6	1444.5	0.3
1813.2	2331.1	2613.1	2472.1	1806.1	0.2
2192.4	2818.2	3074.9	2946.5	2165.3	0.9
2547.8	3286.3	3612.8	3449.5	2546.2	0.1
2923.3	3766.7	4156.4	3961.5	2933.9	−0.4
218.2	377.0	339.2	358.1	205.3	0.4
364.7	553.7	565.0	559.3	357.7	0.2
731.8	977.3	1088.5	1032.9	716.3	0.5
1100.9	1418.4	1612.1	1515.2	1081.5	0.7
1457.2	1855.6	2100.6	1978.1	1432.0	0.8
1817.2	2315.8	2600.9	2458.3	1795.7	0.7
2190.4	2787.4	3073.3	2930.3	2153.1	1.3
2552	3260.9	3617.6	3439.2	2538.4	0.5
2926.5	3725.9	4149.8	3937.8	2916.0	0.4

## Data Availability

The data used or analyzed in this study were obtained from the corresponding authors upon reasonable request.
